# The effects of aerobic exercise in patients with cancer-related fatigue: A systematic review and meta-analysis

**DOI:** 10.1371/journal.pone.0325100

**Published:** 2025-06-09

**Authors:** Tong Wang, Jiaxin Deng, Weicheng Li, Qiubo Zhang, Haoming Yan, Yongfeng Liu

**Affiliations:** 1 School of Sports Training, Chengdu Sport University, Chengdu, Sichuan, China; 2 Primary School Attached to Chengdu Normal College, Chengdu, Sichuan, China; Kamuzu University of Health Sciences (KUHeS), MALAWI

## Abstract

**Background:**

Cancer-related fatigue (CRF) is characterized by an unusual and persistent sensation of tiredness that can occur during or after treatment, potentially impacting both physical and mental capability, and which does not ameliorate with rest. Aerobic exercise (AE) has been identified as a potent modality to mitigate the severity of CRF in such patients.

**Objective:**

This study aims to investigate the efficacy of AE in alleviating CRF among patients.

**Methods:**

A comprehensive literature search was implemented on PubMed, Web of Science, EBSCO, Cochrane, and Embase until June 2024. Studies were selected based on the following PICOS criteria: Participants (P): cancer patients undergoing treatment or in recovery; Intervention (I): aerobic exercise, including activities such as walking, running, yoga, or tai chi; AE interventions conducted during both treatment and recovery were included. Comparison (C): usual care, no-treatment/wait-list, or attention/activity placebo controls; Outcome (O): cancer-related fatigue (CRF) measured by validated scales; Study design (S): randomized controlled trials (RCTs). The meta-analysis was performed using Review Manager 5.3.

**Results:**

The results indicate that AE exerts a significant impact on CRF, but the heterogeneity is high (SMD = −0.76, 95% CI: −1.30 to −0.22, P < 0.05, I² = 94%). Subgroup analysis revealed that AE interventions lasting at least 12 weeks (SMD = −1.12, 95% CI = −2.02 ~ −0.22, P < 0.05, I² = 96%), 3 times or less per week (SMD = −1.00, 95% CI = −1.83 ~ −0.16, P < 0.05, I^2^ = 96%), with each session exceeding 60 minutes (SMD = −1.48, 95% CI = −2.32 ~ −0.64, P < 0.01, I^2^ = 96%), compared to the control group, significantly improve CRF in patients.

**Conclusion:**

The research findings confirm the effectiveness of AE in alleviating CRF. Subgroup analysis further elucidated that AE interventions lasting at least 12 weeks, 3 times or less per week, with 60 minutes or more per session, significantly alleviated CRF among patients. However, given the limited number of included studies, caution is warranted in interpreting these subgroup analysis outcomes. The protocol for this review was duly registered on PROSPERO under the registration number CRD42024559098.

## Introduction

Globally, an estimated 10 million individuals are diagnosed with cancer annually, with an additional 25 million surviving the disease [1]. Although therapeutic advances have prolonged survival, many patients suffer from a myriad of treatment-associated side effects, including the high frequency of individual symptoms and their overall severity [2,3]. Among these adverse effects, cancer-related fatigue (CRF) stand out as a particularly prevalent and debilitating symptoms, resulting in functional decline and diminished quality of life. Furthermore, decline in physical activity levels during therapy and lingering consequences of therapeutic interventions further impair patients’ physical performance and capacity for exercise [4,5].

CRF is defined as “a distressing, persistent, and subjective experience of physical, emotional, and/or cognitive fatigue or exhaustion associated with cancer or its treatment, which is disproportionate to recent activity and impedes normal functioning” [6]. In addition, this symptom significantly impacts daily activities, social interactions, societal reintegration, and overall quality of life [7]. Evidence also suggests that CRF may serve as a prognostic indicator of survival in cancer patients. Fatigue acts as the principal feature of CRF, which ranges from tiredness to profound exhaustion and is unrelated to physical activity. If the intensity of fatigue is disproportionate to physical exertion, unrelieved by rest, or even exacerbated after rest, it may also manifest post-activity [8]. One potential contributor to CRF is energy imbalance [9], which is associated with decreased muscle biosynthesis [10]. These deficiencies are notably sensitive to neuromuscular assessments of skeletal muscle endurance [11].

Aerobic exercise (AE) can be referred to as continuous, rhythmic physical activity that engages large muscle groups and primarily relies on the oxidative (aerobic) energy system to fulfill the body’s energy demands [12]. The mechanisms underlying the impact of AE on CRF may encompass various practices such as traditional Iyengar yoga, which enhances physical function, ultimately alleviating fatigue and improving emotional well-being. This effect may be mediated through decreased cortisol secretion and improved diurnal cortisol rhythm. Alternatively, yoga can potentially modulate stress responses, leading to a reduction in allostatic load and cortisol secretion, thus benefiting fatigue and emotional health [13]. Another plausible mechanism involves multiple simultaneous physiological adaptations within the brain. Exercise that diminishes fatigue is associated with decreased levels of pro-inflammatory cytokines like interleukin-6 and other inflammatory markers, suggesting potential alternations in neurons or microglia in the brain [14,15]. Furthermore, exercise increases peripheral levels of myokines, including cathepsin B, which can traverse the blood-brain barrier and contribute to elevated brain-derived neurotrophic factor (BDNF), thereby enhancing energy levels and reducing fatigue [16]. Additionally, exercise elevates brain concentrations of norepinephrine and dopamine while modulating the density of neurotransmitter receptors in brain regions related to energy and fatigue perception, such as the prefrontal cortex, striatum, and nucleus accumbens [17,18]. These biological adaptations hold promise for improving energy levels and alleviating fatigue but are part of a complex network of interacting neural pathways that are not yet fully comprehended [19].

Exercise interventions have been proven to have a significant effect in relieving CRF during cancer treatment or during rehabilitation post-treatment [20–22]. Typical examples include walking, jogging, cycling, and swimming, which, when performed at an appropriate intensity and duration, augment cardiovascular function and endurance. Exercise training represents the most extensively researched non-pharmacological intervention for managing CRF [23]. Among various cancer survivor cohorts, AE has demonstrated substantial potential in ameliorating CRF [24]. Compared to resistance exercise, AE is more effective in mitigating CRF [25,26]. Activities such as yoga, tai chi, qigong, walking, and running are also beneficial in alleviating CRF. For instance, a study examining a home-based yoga intervention with 32 participants, conducted five times a week for 24 weeks, found that women in the yoga group experienced a remarkably greater reduction in CRF compared to a wait-list control group of 31 participants [27].

This systematic review and meta-analysis aims to rigorously assess the effects of AE on CRF in patients. Our review contributes to the existing literature through analyzing new studies not included in previous systematic reviews and performing additional subgroup analyses (e.g., intervention time, frequency, and duration). This approach clarifies how specific parameters of AE might yield the greatest reduction in CRF.

## Methods

This review followed the Preferred Reporting Items for Systematic Reviews and Meta-Analyses (PRISMA) guidelines [28] and the Cochrane Handbook for Systematic Reviews and Meta-Analysis [29]. In addition, the protocol for this review was duly registered on PROSPERO under the registration number CRD42024559098.

### Search strategy

We systematically searched five databases—Web of Science, EBSCO, PubMed, Cochrane, and Embase—to find relevant literature on June 7, 2024. A summary of the search terms is provided in Table 1. We used the primary search terms “exercise,” “cancer,” “fatigue,” and “randomized controlled trials,” which were combined using Boolean operators (AND and OR). The search strategy was a Boolean logic search with the following search strategies: (Exercise OR “Physical activity” OR “Physical exercise” OR sport OR Training OR Aerobic OR Resistance OR Strength OR “Chronic exercise” OR “Acute exercise”) AND (Cancer) AND (Fatigue OR Lassitude OR Energy OR Vigor OR Vitality) AND (“Randomized controlled trial” OR RCT). Two researchers (TW and WCL) independently assessed the titles and abstracts of the remaining articles. Any study considered relevant by either researcher moved forward to full-text screening. For full-text screening, the same two researchers (TW and WCL) independently evaluated each article against our predefined inclusion and exclusion criteria. Discrepancies—at either the title/abstract stage or the full-text stage—were resolved through discussion. If disagreement persisted, a third researcher (YFL) was consulted to reach a consensus. Finally, the data were analyzed by TW, HMY, and JXD and supervised and reviewed by QBZ and YFL.

**Table 1:** Summary of search terms.

Category		Included search terms
Exercise		(Exercise OR “Physical activity” OR “Physical exercise” OR sport OR Training OR Aerobic OR Resistance OR Strength OR “Chronic exercise” OR “Acute exercise”)
	AND	
Cancer		(Cancer)
	AND	
Fatigue		(Fatigue OR Lassitude OR Energy OR Vigor OR Vitality)
	AND	
Randomized controlled trial		(Randomized controlled trial OR RCT)

### Eligibility criteria

The inclusion criteria for relevant studies were established based on the PICOS framework. Participants (P): We included adult patients (≥18 years old) with a confirmed cancer diagnosis, whether the patient was undergoing treatment or in recovery. We did not restrict by cancer type (e.g., breast, prostate, colorectal), as our objective was to examine AE effects on CRF broadly. Intervention (I): Our review focused on studies where the primary or adjunct intervention was AE. This encompassed walking, running, cycling, aquatic exercise, and/or group classes such as tai chi or yoga (as long as the study explicitly described the intervention’s aerobic component). Comparison (C): We included randomized controlled trials featuring any of the following comparator arms: No-treatment or wait-list control (NT/WL), usual care (UC), or attention or activity placebo (AP). Outcome (O) measures focused on CRF. Subgroup analyses were conducted when instruments were used in more than one study. Study design (S): We restricted our inclusion to randomized controlled trials (RCTs), published in English, that reported sufficient data (e.g., means, standard deviations, sample sizes) for effect-size calculation. We excluded conference abstracts, case studies, and other non-RCT designs. Additionally, studies not in English, including unpublished materials, theses, reviews, those involving animals, those with insufficient data for extraction, duplicate publications, and studies with inaccessible full texts, were excluded.

### Data extraction

References were managed and screened using EndNote 20 software, and detailed data extraction was independently performed by two researchers (TW and WCL) using structured data extraction forms in Excel, with a third researcher (YFL) consulted in case of disagreement. Data were analyzed by TW, WCL, and JXD and supervised and reviewed by QBZ and YFL. Data from both the intervention and control groups, including mean values, standard deviations, and participant counts, were entered into Review Manager 5.3 [29]. Given the expected variability in aerobic interventions across trials, a meta-analysis was performed using a random-effects model to account for potential heterogeneity. To synthesize data from various fatigue scales, the effect size was assessed as the standardized mean difference (SMD) using Hedges’ g, which adjusts for small sample size bias, and reported with 95% confidence intervals (CI) [30]. In addition to the overall meta-analysis across all validated fatigue scales, we conducted separate meta-analyses for each individual measurement tool whenever two or more studies used that same instrument (e.g., MFI, MFSI, FACT, FACIT, PFS, CFS, BFI, EORTC QLQ-FA13). Heterogeneity was evaluated using the I² statistic [29]. Significant heterogeneity (I² > 50%) led to subgroup or sensitivity analyses to elucidate the findings [31]. Sensitivity analyses were conducted by excluding individual studies from the analysis on a case-by-case basis to assess their impact on the overall results to determine their potential effect on heterogeneity. If the results obtained after each exclusion remained consistent with the results of the preliminary analysis, it was concluded that no single study had a significant impact on the final results. Subgroup analyses were conducted based on the type of control group, type of cancer, timing of intervention, and instruments used to assess CRF. The objectives of these subgroup analyses included the type of control group (WL/NT, AP, UC), the intensity of AE intervention (high, moderate), the duration of the activity intervention (<8 weeks, 811 weeks, ≥ 12 weeks), the time of each activity session (<60 minutes, ≥ 60 minutes), and the frequency of the activity intervention (≤3 times/week, > 3 times/week). According to the American College of Sports Medicine (ACSM) criteria [32], high-intensity exercise can be categorized according to different indicators: 60%−89% of HRR, 77%−95% of HRmax, 15–17 points of RPE, and 64%−90% of VO_2peak_, while moderate-intensity corresponds to 40%−59% HRR, 64%−76% HRmax, 12–14 points of RPE, and 46%−63% VO_2peak_.

### Methodological quality assessment

We evaluated the methodological quality of each study using the Physiotherapy Evidence Database (PEDro) scale [33], a reliable and validated tool for assessing RCTs. The PEDro scale comprises 11 criteria related to eligibility, randomization, allocation, and blinding [34]. Scores range from 0 to 10. Previous research has indicated that blinding in AE intervention trials is often not feasible. Consequently, ensuring blinding of participants and therapists in physical activity interventions and obtaining dual scores can be challenging [35]. To address the inherent limitations of AE interventions, we segmented the scoring system into three distinct categories, consistent with prior assessments. Specifically, a score of 6 or higher was considered indicative of high-quality studies, reflecting rigor and comprehensiveness. Scores between 4 and 5 were classified as moderate quality, indicating acceptable standards in research methodology. A score of 3 or lower suggested low quality, pointing to areas for improvement in the study design. This classification scheme allows for a precise evaluation of intervention quality and offers valuable insights for future research.

## Results

### Study selection

**Fig 1:**
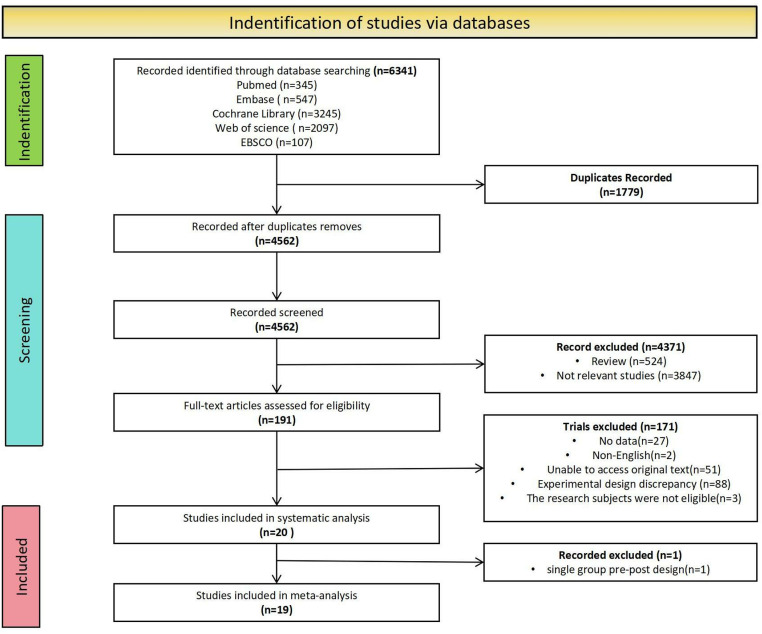
Flow chart of literature retrieval.

The results of the review search and the study selection process are presented in **Fig 1**. Initially, 6,341 articles were retrieved from the database. After removing duplicates, 4,562 studies remained. At the title and abstract screening stages, 4,371 studies were excluded for not meeting the eligibility criteria. Of the 191 studies, 171 were excluded after reading the full text: no data (n = 27); non-English (n = 2); unable to access original text (n = 51); experimental design discrepancy (n = 88); the research subjects were not eligible (n = 3). A systematic analysis identified 20 articles. After excluding a single-group pre-post design, 19 articles were included in the meta-analysis.

### Study characteristics

The characteristics of the included studies are detailed in **Table 2**. The 19 meta-analyzed studies encompassed a total sample size of 1,155 participants, with 568 in the intervention group and 587 in the control group. The age of participants ranged from 18 to 93 years and included various cancer types, such as breast cancer (BC), colorectal cancer (CC), prostate cancer (PC), lung cancer (LC), testicular cancer (TC), differentiated thyroid cancer (DTC), and nasopharyngeal cancer (NC). Intervention modalities included walking, cycling, aquatic sports, yoga, and Baduanjin. The interventions varied in intensity from moderate to high, with durations ranging from 1 to 72 weeks, frequencies from 2 to 7 sessions per week, and session durations from 15 to 90 minutes. The outcome is a CRF, and the instrument used to measure the outcome included the MFI (n = 1), MFSI (n = 4), FACT (n = 4), FACIT (n = 2), PFS (n = 3), CFS (n = 2), BFI (n = 1), POMS (n = 1) and EORTC QLQ-FA13 (n = 1). The comparison groups consisted of no treatment (NT) (n = 3), usual care (UC) (n = 8), waitlist (WL) (n = 3), and an attention/activity placebo (AP) group (n = 5). AE interventions conducted during both treatment and recovery were included.

**Table 2:** Descriptive characteristics of included studies.

Included studies	Sample size (IG/CG)	Age (IG/CG)	Gender	Type of Cancer	Intervention	Intensity	Duration/Time/Frequency (weeks/min/times)	Measurement tools for outcome indicators	Control group	Treatment or rehabilitation stage
Adams, S., 2018^a^	26/35	IG:44.0 ± 11.6 CG:43.3 ± 9.9	Men	TC	HIIT	High (75% to 95% of VO_2peak_)	12/35/3	FACT-F	UC	Undergoing treatment
Adams-Campbell, L. L., 2023^a^	15/15	IG:63.3 ± 3.2 CG:64.5 ± 3.2	Women	BC	Pedlar	Moderate (Moderate)	8/15/5	FACIT-F	NT	Undergoing treatment
Banasik, J., 2011^a^	7/ 7	IG:63.33 ± 6.9 CG:62.4 ± 7.3	Women	BC	Lyengar Yoga	Unreported	8/90/2	FACT-B	NT	Undergoing treatment
Baruth, M., 2015^a^	12/20	IG:57.4 ± 6.1 CG:54.9 ± 6.5	Women	BC	Home-Based Walking	Moderate to Vigorous (RPE 12–15)	12/20-40/3-5	FACT-F	UC	Undergoing treatment
Campo, R. A., 2014^a^	16/13	72(58–93)	Men	PC	Qigong	High (Somewhat strong)	12/40/2	FACT-F	AP	Undergoing treatment
Cantarero-Villanueva, I., 2013^a^	34/34	IG:47 ± 8 CG:49 ± 7	Women	BC	Aquatic program	Unreported	8/60/3	PFS	UC	Undergoing treatment
Chan, M. L. T., 2022^a^	61/63	IG:77.2 ± 7.3 CG:78.1 ± 7.4	Women Men	Multimorbidity	Stepping	Moderate (RPE 12–14)	12/60/3	MFI-20	AP	Undergoing treatment
Cohen, J., 2021^a^	14/13	IG:59.71 ± 6.99 CG:58.56 ± 10.41	Women	BC	Stationary cycling	Moderate (Moderate)	1/20/3	PFS	AP	Undergoing treatment
Dimeo, F. C., 1999^a^	27/32	IG:40 ± 11 CG:40 ± 10	Women Men	Solid tumors or lymphomas	Biking	Moderate (50%HRR)	NR/20/7	POMS	UC	Undergoing treatment
Dimeo, F. C., 2004^a^	27/42	IG:55.1 ± 10 CG:60 ± 9.5	Women Men	LC	Biking	High (80%HRmax)	3/30/5	EORTC QLQ-FA13	AP	Rehabilitation stage
Kiecolt-Glaser, J. K., 2014^a^	96/90	IG:51.8 ± 9.8 CG:51.3 ± 8.7	Women	BC	Yoga	Unreported	12/90/2	MFSI-SF	WL	Undergoing treatment
Lu, Y., 2019^a^	43/44	IG:55.60 ± 11.23 CG:54.63 ± 11.88	Women Men	CC	Baduanjin	Unreported	24/20-40/5	BFI	UC	Undergoing treatment
Lundt, A., 2019	58	58.19 ± 11.97	Women Men	Tumor entity	Yoga	Unreported	8/60/5	EORTC QLQ-FA13	–	Undergoing treatment
Monga, U., 2007^a^	11/10	IG:68 ± 4.2 CG:70.6 ± 5.3	Men	PC	Walking	Moderate (65%HRmax)	8/40/3	PFS	UC	Undergoing treatment
Murley, B.,2019^a^	3/3	49.8 ± 14.5	Women	Solid-tumor	Tai Chi	Unreported	8/50/3	FACIT-F	WL	Undergoing treatment
Schad, F., 2023^a^	24/28	IG:61.7 ± 9.4 CG:59.3 ± 11.0	unreported	BC	Tango	Unreported	6/60/6	CFS	WL	Undergoing treatment
Sprod, L. K., 2015^a^	53/44	IG:67.91 ± 1.05 CG:64.81 ± 0.59	Women Men	BC; Others	Yoga	Unreported	4/75/2	MFSI-SF	UC	Rehabilitation stage
Vigário, P. D. S., 2011^a^	19/17	IG:48.00 CG:50.50	Women Men	DTC	Walking or Running	Moderate (65% and 75% of HRmax)	12/60/2	CFS	NT	Undergoing treatment
Zhang, L. L., 2016^a^	38/36	18-60	Women Men	LC	Tai Chi	Unreported	12/60/3	MFSI-SF	AP	Undergoing treatment
Zhou, W., 2018^a^	42/41	18-70	Women Men	NC	Tai Chi	Unreported	72/60/5	MFSI-SF	UC	Undergoing treatment

AP: Attention/Activity Placebo; BC: Breast Cancer; BFI: Brief Fatigue Inventory; CC: Colorectal Cancer; CG: Comparison Group; CFS: Cancer Fatigue Scale; DTC: Differentiated Thyroid Carcinoma; EORTC QLQ-FA13: European Organization for Research and Treatment of Cancer Quality of Life Questionnaire–Fatigue Scale; FACT-B: Quality of Life Breast Cancer Specific Questionnaire; FACIT-F: Functional Assessment of Chronic Illness Therapy; FACT-F: Functional Assessment of Cancer Therapy Fatigue Scale; HIIT: High-intensity interval training; HRR: Heart Rate Reserve; HRmax: Maximum Heart Rate; IG: Intervention Group; PC: Prostate Cancer; PFS: Piper Fatigue Scale; RPE: Rating of Perceived Exertion; LC: Lung cancer; MFI: Multidimensional Fatigue Inventory; MFSI: Multidimensional Fatigue Symptom Inventory-Short Form; NC: Nasopharyngeal Carcinoma; NT: No treatment; TC: Testicular Cancer; UC: Usual Care;, VO2peak = volume of oxygen consumption at peak exercise.; WL: Wait-List

^a^Studies included in the meta-analysis

### Quality assessment

**Table 3** evaluates the methodological rigor of the studies in our analysis, all of which met at least three essential criteria, ensuring a basic level of scientific rigor. Twelve studies exhibited high methodological quality, with an average quality score of 5.9 across the meta-analysis, reflecting a solid methodological foundation. Recruitment criteria were clearly defined in each study, ensuring appropriate participant selection. High retention rates minimized attrition, preserving data integrity. However, few studies effectively used blinding procedures, with only four adequately blinding outcome assessors, which is essential for reducing bias and enhancing the reliability of the findings.

**Table 3:** Methodological quality assessment for included studies.

Included studies	Eligibility Criteria	Random Allocation	Allocation Concealment	Similar at Baseline	Subject Blinded	Therapist Blinded	Assessor Blinded	Dropout Rate	Intention-to-treat Analysis	Between-group Comparison	Points Measures	Total score	Overall Study Quality
Adams, S., 2018^a^	1	1	1	1	0	0	0	1	0	1	1	6	High
Adams-Campbell, L. L., 2023^a^	1	1	0	1	0	0	0	1	0	1	1	5	Adequate
Banasik, J., 2011^a^	1	1	0	1	0	0	0	1	0	1	1	5	Adequate
Baruth, M., 2015^a^	1	1	0	1	0	0	0	1	0	1	1	5	Adequate
Campo, R. A., 2014^a^	1	1	1	1	0	0	1	1	0	1	1	7	High
Cantarero-Villanueva, I., 2013^a^	1	1	1	1	0	0	0	1	0	1	1	6	High
Chan, M. L. T., 2022^a^	1	1	1	1	0	0	0	1	0	1	1	6	High
Cohen, J., 2021^a^	1	1	1	1	0	0	0	1	0	1	1	6	High
Dimeo, F. C., 1999^a^	1	1	0	1	0	0	0	1	0	1	1	5	Adequate
Dimeo, F. C., 2004^a^	1	1	1	1	0	0	0	1	0	1	1	6	High
Kiecolt-Glaser, J. K., 2014^a^	1	1	1	1	0	1	1	1	0	1	1	8	High
Lu, Y., 2019^a^	1	1	1	1	0	0	0	1	0	1	1	6	High
Lundt, A., 2019	1	0	0	1	0	0	0	1	0	0	1	3	Poor
Monga, U., 2007^a^	1	1	0	1	0	0	0	1	0	1	1	5	Adequate
Murley, B.,2019^a^	1	1	0	1	0	0	0	1	0	1	1	5	Adequate
Schad, F., 2023^a^	1	1	1	1	1	1	1	1	0	1	1	9	High
Sprod, L. K., 2015^a^	1	1	1	1	0	1	1	1	0	1	1	8	High
Vigário, P. D. S., 2011^a^	1	1	0	1	0	0	0	1	0	1	1	5	Adequate
Zhang, L. L., 2016^a^	1	1	1	1	0	0	0	1	0	1	1	6	High
Zhou, W., 2018^a^	1	1	0	1	0	0	0	1	1	1	1	6	High

*Yes = 1; No = 0;

≤3 are considered ‘poor’; 4–5 are considered ‘adequate’; ≥ 6 are high.

^a^Studies included in meta-analysis

### Sensitivity analysis

To further explore sources of heterogeneity, we conducted a sensitivity analysis. These analyses considered factors such as study design, sample size, and quality scores to determine their potential impact on heterogeneity. The results obtained after each exclusion were consistent with the results of the initial analyses, indicating that none of the studies had a significant impact on the pooled results. This consistency suggests that the composite effect size in this study is stable and robust.

### Mata-analysis

**Fig 2:**
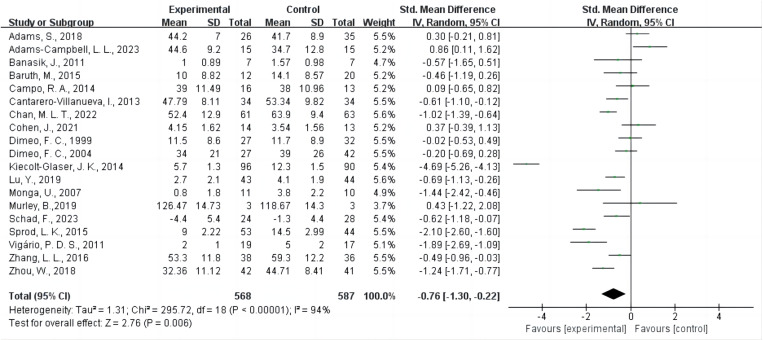
Forest plot of the effect of AE on CRF. Green squares indicate each study’s point estimate, with the size of the square proportionate to that study’s weight in the meta-analysis. Horizontal lines through each square represent the 95% confidence intervals (CIs). The diamond at the bottom depicts the pooled SMD and 95% CI. The solid vertical line at SMD = 0 represents no difference between groups. A random-effects model was employed to account for heterogeneity among the included studies. The test for the overall effect suggests a statistically significant reduction in CRF with AE compared to control. Abbreviations: AE, aerobic exercise; CRF, cancer-related fatigue; SMD, standardized mean difference; CI, confidence interval.

The results indicate that AE significantly reduces CRF (Fig 2), despite high heterogeneity (SMD = −0.76, 95% CI: −1.30 to −0.22, P = 0.006 < 0.01, I² = 94%). AE significantly reduced CRF as measured by MFI [36] and MFSI [22,37–39] (SMD = −1.90, 95% CI: −3.20 to −0.61, P = 0.004 < 0.01, I² = 97%), and CFS [40,41] (SMD = −1.22, 95% CI: −2.46 to 0.55, P = 0.05, I² = 84%). However, AE did not significantly affect outcomes when using FACT [13,20,42,43] and FACIT [21,44] (SMD = 0.13, 95% CI: −0.30 to 0.55, P = 0.56, I² = 40%), or PFS [45–47] (SMD = −0.52, 95% CI: −1.40 to 0.36, P = 0.24, I² = 77%) as outcome measures.

### Subgroup analysis

The results of the subgroup analyses are shown in Table 4.

**Table 4:** Subgroup analyses based on the primary meta-analysis.

Subgroup analysis	K	N(IG/CG)	SMD	95%CI	P value	Heterogeneity	Test for subgroup differences
Primary meta-analysis	19	568/587	−0.76	−1.30 to −0.22	**P** = 0.006** < 0.01**	X^2 ^= 295.72, df = 18 (P < 0.00001), I^2 ^= 94%	
Type of control group							
AE v. NT/WL	6	164/160	−1.11	−3.00 to 0.78	P = 0.25	X^2 ^= 178.89, df = 5 (P < 0.00001), I^2 ^= 97%	X² = 1.84, df = 2, P = 0.006, I² = 0%
AE v. AP	5	156/167	−0.32	−0.80 to 0.16	P = 0.19	X^2 ^= 16.16, df = 4 (P = 0.003), I^2 ^= 75%	
AE v. UC	8	248/260	−0.77	−1.32 to −0.21	**P = 0.007 < 0.01**	X^2 ^= 59.06, df = 7 (P < 0.00001), I^2 ^= 88%	
Intensity							
High	3	69/90	0.05	−0.27 to 0.36	P = 0.78	X^2 ^= 2.01, df = 2 (P = 0.37), I^2 ^= 0%	X² = 1.70, df = 1, P = 0.25, I² = 41.2%
Moderate	6	147/150	−0.51	−1.27 to 0.26	P = 0.20	X^2 ^= 43.02, df = 5 (P < 0.00001), I^2 ^= 88%	
Duration							
<8 weeks	4	118/127	−0.66	−1.70 to 0.38	P = 0.21	X^2 ^= 41.16, df = 3 (P < 0.00001), I^2 ^= 93%	X² = 1.76, df = 2, P = 0.005, I² = 0%
8-11 weeks	5	70/69	−0.30	−1.11 to 0.51	P = 0.47	X^2 ^= 16.1, df = 4 (P = 0.002), I^2 ^= 76%	
≥12 weeks	9	353/359	−1.12	−2.02 to −0.22	**P = 0.01 < 0.05**	X^2 ^= 213.56, df = 8 (P < 0.00001), I^2 ^= 96%	
Time							
<60 min	10	194/227	−0.09	−0.45 to 0.26	P = 0.61	X^2 ^= 26.27, df = 9 (P = 0.002), I^2 ^= 66%	X² = 8.82, df = 1, P = 0.006, I² = 88.7%
≥60 min	9	374/360	−1.48	−2.32 to −0.64	**P** = 0.0006** < 0.001**	X^2 ^= 180.16, df = 8 (P < 0.00001), I^2 ^= 96%	
Frequency							
≤3 times/week	12	378/365	−1.00	−1.83 to −0.16	**P = 0.02 < 0.05**	X^2 ^= 245.24, df = 11 (P < 0.00001), I^2 ^= 96%	X² = 1.67, df = 1, P = 0.006, I² = 40.2%
>3 times/week	7	190/222	−0.38	−0.82 to 0.06	P = 0.09	X^2 ^= 27.67, df = 6 (P < 0.00001), I^2 ^= 78%	

AE: Aerobic exercise; K: Number of trials; N: Number of participants; SMD: Standardized Mean Difference; CI: Confidence Interval; NT: No treatment; AP: Attention/Activity Placebo; UC: Usual Care; WL: Wait-List.

### Type of control group

**Fig 3:**
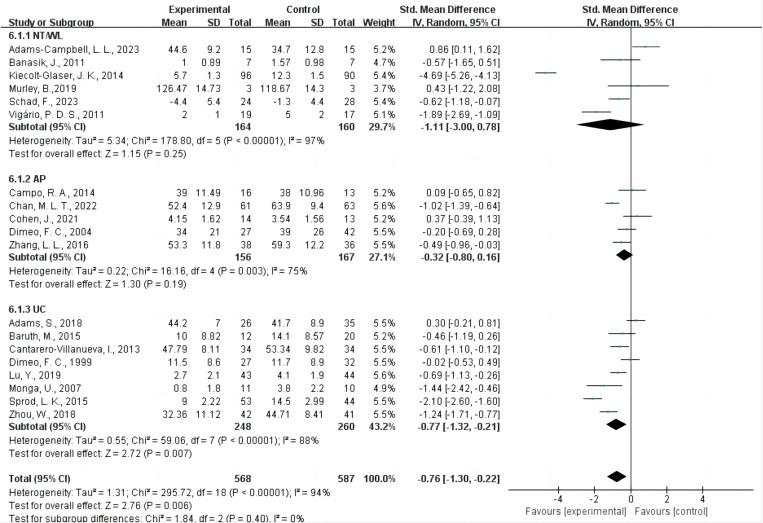
Forest plot of the effect of type of control group on CRF. Green squares represent individual study effect estimates (SMDs), with the size of each square proportional to the study’s weight. Horizontal lines indicate 95% confidence intervals (CIs). Diamonds show pooled effect sizes for each subgroup and for the overall meta-analysis. The vertical line at SMD = 0 marks no difference between AE and the respective control. A random-effects model was used, accounting for heterogeneity. The test for overall effect suggests a statistically significant benefit of AE compared with control for reducing CRF. Abbreviations: AE, aerobic exercise; CRF, cancer-related fatigue; SMD, standardized mean difference; CI, confidence interval.

The control group type in six studies [13,21,37,40,41,44] was NT/WL, in five studies [36,38,43,46,48] was AP, and in eight studies [20,22,39,42,45,47,49,50] was UC (Fig 3). Compared to the UC group, AE was associated with a significant improvement in CRF (SMD = −0.77, 95% CI = −1.32 to −0.21, P = 0.007, I² = 88%). In contrast, AE did not result in significantly better CRF outcomes when compared with the NT/WL group (SMD = −1.11, 95% CI = −3.00 to 0.78, P = 0.25, I² = 97%) or the AP group (SMD = −0.32, 95% CI = −0.80 to 0.16, P = 0.19, I² = 75%).

### Intensity of AE intervention

**Fig 4:**
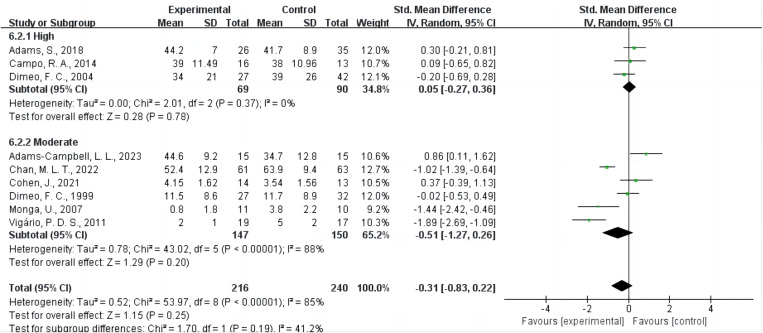
Forest plot of the effect of AE intensity on CRF. Green square denotes an individual study’s effect size (SMD), with the square’s size reflecting the study’s weight in the meta-analysis. Horizontal lines represent 95% confidence intervals (CIs). Diamonds show the pooled estimates for each subgroup (high or moderate intensity) and the overall effect across both subgroups. The vertical line at SMD = 0 corresponds to no difference in CRF between exercise and control groups. A random-effects model was used to account for heterogeneity. The overall effect size indicates a nonsignificant reduction in CRF favoring AE compared with control. Abbreviations: AE, aerobic exercise; CRF, cancer-related fatigue; SMD, standardized mean difference; CI, confidence interval.

Ten studies did not explicitly report exercise intensity based on standard physiological metrics. Consequently, these studies were categorized as having an “unreported” intensity in **Table 2**. Three studies [20,43,48] involved high-intensity interventions, six studies [21,36,41,46,47,49] involved moderate-intensity interventions, and none involved low-intensity interventions (Fig 4). The results indicated that neither high-intensity AE (SMD = 0.05, 95% CI = −0.27 ~ 0.36, P = 0.78, I² = 0%) nor moderate-intensity AE (SMD = −0.51, 95% CI = −1.27 ~ 0.26, P = 0.20, I² = 88%) improved CRF.

### Duration of AE intervention

**Fig 5:**
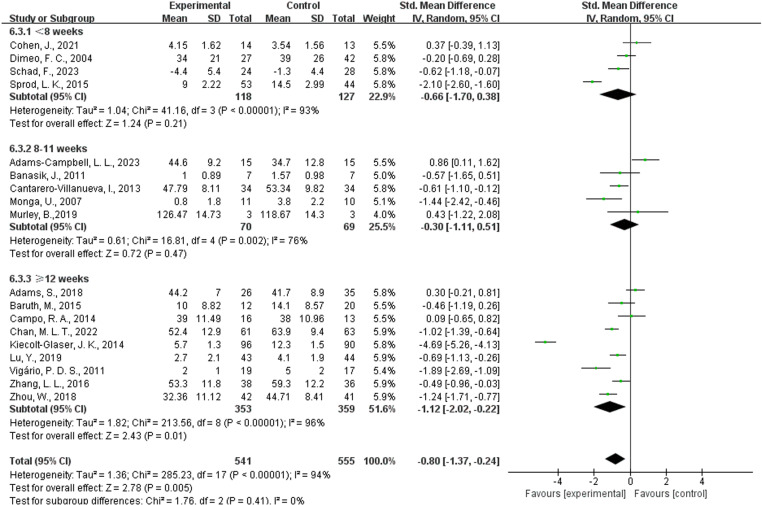
Forest plot of the effect of AE duration on CRF. Green squares represent individual study effect estimates, with square size proportional to each study’s weight. Horizontal lines denote 95% confidence intervals (CIs). Diamonds display pooled effect estimates and CIs for each subgroup and for the overall analysis. The vertical line at SMD = 0 marks the point of no difference between AE and control. A random-effects model was used to account for heterogeneity. The overall effect size indicates a statistically significant reduction in CRF with AE compared to control. Abbreviations: AE, aerobic exercise; CRF, cancer-related fatigue; SMD, standardized mean difference; CI, confidence interval.

Four studies [22,40,46,48] provided data on interventions lasting less than 8 weeks; five studies [13,21,44,45,47] provided data on interventions lasting 8–11 weeks; and nine studies [20,36–39,41–43,50] provided data on interventions lasting 12 weeks or more (Fig 5). The results indicated that when the intervention lasted at least 12 weeks (SMD = −1.12, 95% CI = −2.02 ~ −0.22, P = 0.01 < 0.05, I² = 96%), AE interventions had a significant impact on CRF, though heterogeneity was high. For interventions lasting less than 8 weeks (SMD = −0.66, 95% CI = −1.70 ~ 0.38, P = 0.21, I² = 93%) and those lasting 8–11 weeks (SMD = −0.30, 95% CI = −1.11 ~ 0.51, P = 0.47, I² = 76%), AE interventions did not have a significant impact on CRF.

### Duration of each session in the AE intervention

**Fig 6:**
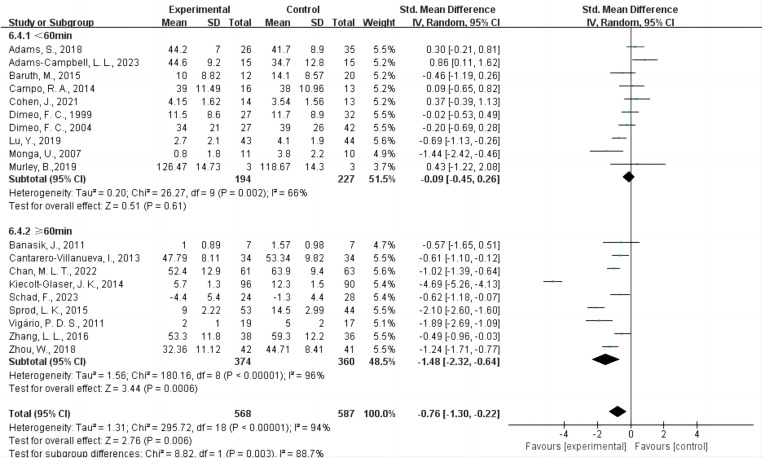
Forest plot of the effect of AE time on CRF. Green squares denote individual study estimates (SMDs), with the size of each square reflecting the study’s relative weight in the meta-analysis. Horizontal lines mark 95% confidence intervals (CIs). Diamonds display the pooled effect sizes for each subgroup and the overall analysis. The vertical line at SMD = 0 signifies no difference between AE and control. A random-effects model was used to account for heterogeneity. The overall pooled effect indicates a statistically significant reduction in CRF with AE. Abbreviations: AE, aerobic exercise; CRF, cancer-related fatigue; SMD, standardized mean difference; CI, confidence interval.

Ten studies [20,21,42–44,46–50] provided data on intervention sessions lasting less than 60 minutes, while nine studies [13,22,36–41,45] provided data on sessions lasting 60 minutes or more per session (Fig 6). The results showed that when the intervention sessions lasted 60 minutes or more per session (SMD = −1.48, 95% CI = −2.32 ~ −0.64, P = 0.0006 < 0.001, I² = 96%), AE intervention had a significant impact on CRF. In contrast, when the sessions lasted less than 60 minutes (SMD = −0.09, 95% CI = −0.45 ~ 0.26, P = 0.61, I² = 66%), AE intervention did not have a significant impact on CRF.

### Frequency of AE intervention

**Fig 7:**
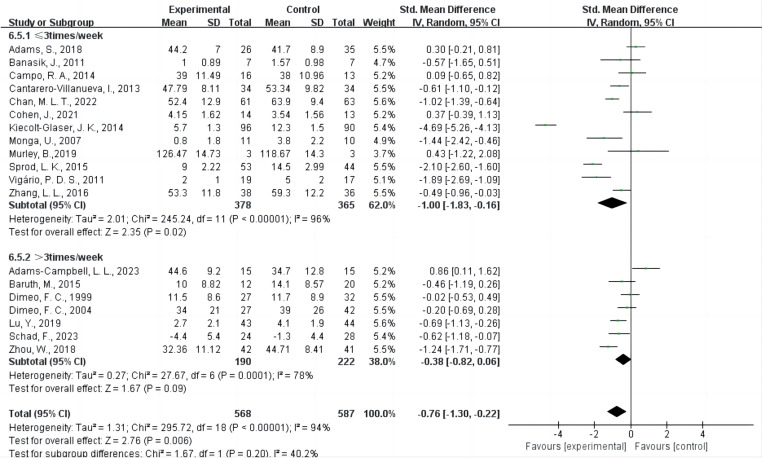
Forest plot of the effect of AE frequency on CRF. Green squares represent individual study SMDs, with square size reflecting each study’s weight in the meta-analysis. Horizontal lines show 95% confidence intervals (CIs). Diamonds represent pooled effect sizes for each frequency subgroup and for the overall analysis. The vertical line at SMD = 0 marks no difference between AE and control. A random-effects model was applied to account for heterogeneity. The overall pooled effect supports a beneficial impact of AE in reducing CRF. Abbreviations: AE, aerobic exercise; CRF, cancer-related fatigue; SMD, standardized mean difference; CI, confidence interval.

Twelve studies [13,20,22,36–38,41,43–47] provided data on interventions conducted three times per week or less, while seven studies [21,39,40,42,48–50] provided data on interventions conducted more than three times per week (Fig 7). The results indicated that AE interventions conducted three times per week or less (SMD = −1.00, 95% CI = −1.83 ~ −0.16, P = 0.02 < 0.05, I² = 96%) significantly improved CRF. In contrast, AE interventions conducted more than three times per week (SMD = −0.38, 95% CI = −0.82 ~ 0.06, P = 0.09, I² = 78%) did not have a significant impact on improving CRF.

## Discussion

The objective of this study is to investigate the impact of AE on CRF through a comprehensive systematic review and meta-analysis. Based on the eligibility criteria, 19 studies were included.

### Mata-analysis findings

Our analysis underscores the pronounced efficacy of AE in attenuating CRF [51–53]. The notion that AE improves CRF is further supported by this systematic review [54,55]. AE is strongly associated with improvements in lung function, cardiovascular health, and self-esteem, and may help patients reduce anxiety and depression and promote a return to relatively stable levels of all aspects of health (E.g. CRF) that are typically attenuated in patients undergoing radiotherapy [56]. Mock et al. reported that adherence to moderate-intensity walking exercise at home in the treatment of breast cancer alleviated high levels of CRF [57]. Moreover, in 2014, Sadja et al. reported that yoga functions as a potent method for alleviating CRF in women with BC compared to a non-active control group [58]. A research by Boehm et al. endorsing the efficacy of yoga as a non-pharmacological intervention for CRF management among BC patients [59]. Overall, these converging findings emphasize the necessity of incorporating tailored AE into comprehensive cancer care protocols to effectively alleviate CRF and enhance quality of life.

In the assessment of CRF mitigation, specialized instruments such as the MFI, MFSI, and CFS have consistently captured substantial reductions in CRF severity following AE interventions. Conversely, assessments utilizing broader quality-of-life measures—namely the FACT, FACIT, and PFS—did not uniformly reflect the beneficial impact of AE on CRF. This divergence suggests that instruments like the MFI and MFSI designed to assess specific dimensions of CRF are more sensitive to the nuanced fluctuations induced by exercise interventions; whereas broader measures may inadvertently obscure these intervention effects by encompassing a wider array of general CRF-related experiences. Consequently, these findings highlight the critical importance of carefully selecting measurement instruments in future research. Although FACT-F and FACIT are validated as the best CRF measurement tools [60], the present study demonstrated that FACT-F and FACIT are not as suitable as MFI and MFSI for measuring CRF in cancer patients, which is in line with previous studies [61]. Researchers should explicitly define the targeted dimensions of CRF and standardize evaluation protocols to reduce heterogeneity, thereby optimizing the comparability and interpretability of study outcomes.

### Subgroup analyses findings

Our subgroup analyses included the type of control group, intervention intensity, duration, session length, and frequency. The results suggest that intervention intensity did not substantially modulate its effect. Notably, AE was associated with improved CRF outcomes in studies that used the UC group as the comparator. Further results showed that patients with CRF experienced prominent symptom enhancement after participating in AE lasting longer than 12 weeks and performed up to three times per week for more than 60 minutes per session. However, the results of these subgroup analyses must be interpreted with caution due to the limited number of included studies.

The results showed that AE was associated with improved CRF outcomes in comparisons with the UC group, but did not show significant improvements in comparisons with the NT/WL or AP groups. This outcome aligns with Mohr’s assertion that different control conditions yield varying comparative results due to their inherent characteristics [62]. This conclusion is also consistent with three earlier meta-analyses that evaluated the effects of AE against various non-active reference groups, including control groups, waiting lists, and supportive therapy groups [59,63,64]. UC, as a standard clinical practice, often lacks structured interventions or psychosocial support, rendering AE appear more beneficial in comparison. For instance, a three-month AE intervention (N = 222) improved CRF in BCS compared to UC [65]. In contrast, NT/WL controls—although seemingly inert—may introduce expectancy and behavioral biases that suppress spontaneous improvement, thereby minimizing observed differences with AE. This result can be attributed to the characteristics of the NT/WL. Addtionally, no-treatment controls are particularly susceptible to problems associated with treatment fidelity procedures effects and clinician selection and allegiance biases [62]. AP controls, which involve established interventions, may produce therapeutic benefits similar to AE, thus reducing the likelihood of detecting a statistically significant difference. In a randomized controlled experimental study in which the control group was AP, Dimeo assigned 72 patients undergoing surgery for lung cancer or gastrointestinal tumors to either an AE group or a progressive relaxation training group [48]. The study results revealed that both AE and progressive relaxation techniques are effective therapies for treating post-surgical CRF in cancer patients. These findings reinforce Moh’s argument that control condition selection is not neutral; it shapes both the internal validity and the observed effect size of interventions, requiring careful alignment with study hypotheses and methodological rigor to avoid misinterpretation of outcomes.

The results of our study showed that patients with CRF experienced significant improvement in their symptoms after completing more than 12 weeks of treatment. This observation is consistent with the findings of previous research that corroborates AE’s effectiveness in enhancing CRF, particularly over a minimum of 12 weeks [66]. CRF was diminished in our 12-week intervention, with effects in the small to moderate range. These findings are promising given that AE is a simple, low-cost, low-burden intervention [42]. Both Pinto et al. [67] and Valens et al. [68] found significant reductions in CRF symptoms with the 12-week follow-up (neither study reported effect sizes). Mustian et al. reported enhanced functional capacity in BC survivors following a 12-week Tai Chi program, [69] Collectively, these studies underscore the value of a structured 12-week exercise protocol in mitigating CRF. The findings of this study demonstrate that regarding session duration, our investigation reveals that AE, conducted up to three times weekly, with each session lasting a minimum of 60 minutes, notably enhances CRF. A meta-analysis showed that AE performed 2–3 times per week had a profound impact in alleviating CRF [61]. This observation aligns with recommendations from a prior randomized controlled trial pertaining to the Tai Chi intervention that was administered three times per week for 12 weeks [70]. Moreover, most AE interventions for cancer survivors typically encompass 2–3 sessions per week spanning 10–24 weeks [71]. In summary, subsequent AE interventions lasting at least 12 weeks, 3 times or less per week, with 60 minutes or more per session, significantly alleviated CRF among patients.

### Limitations of the review

This review possesses several limitations. Firstly, it encompasses a restricted number of studies and samples, potentially resulting in inconclusive findings regarding the effectiveness of exercise interventions. Secondly, most studies employed subjective self-report questionnaires to assess CRF in patients. However, this measurement approach can introduce subjectivity and compromise the accuracy and reliability of data collection. Thirdly, the study exclusively examined the effects of AE on CRF, while future research could investigate the influence of resistance exercise or combined exercise regimens on CRF.

## Conclusion

The research findings confirm the effectiveness of AE in alleviating CRF through a systematic review and meta-analysis. CRF was evaluated using subjective measures such as MFSI, MFI, FACT, FACIT, PFS, CFS, BFI, and EORTC QLQ-FA13. Subgroup analysis further elucidated that AE interventions lasting at least 12 weeks, 3 times or less per week, with 60 minutes or more per session, significantly alleviated CRF among patients. However, given the limited number of included studies, caution is warranted in interpreting these subgroup analysis outcomes.

## Supporting information

S1 File Records retrieved for each database. (ZIP)

S2 File PRISMA_2020_checklist. (DOCX)

S1 Table All literature included and excluded and reasons. (XLSX)

S2 Table Study characteristics. (XLSX)

S3 Table Quality assessments. (XLSX)
