# Supplementary figures and images for: The effects of aerobic exercise in patients with cancer-related fatigue: A systematic review and meta-analysis
Source: PLoS One. 2025 Jun 9;20(6):e0325100. doi: 10.1371/journal.pone.0325100 (PMC12148172; doi:10.1371/journal.pone.0325100)

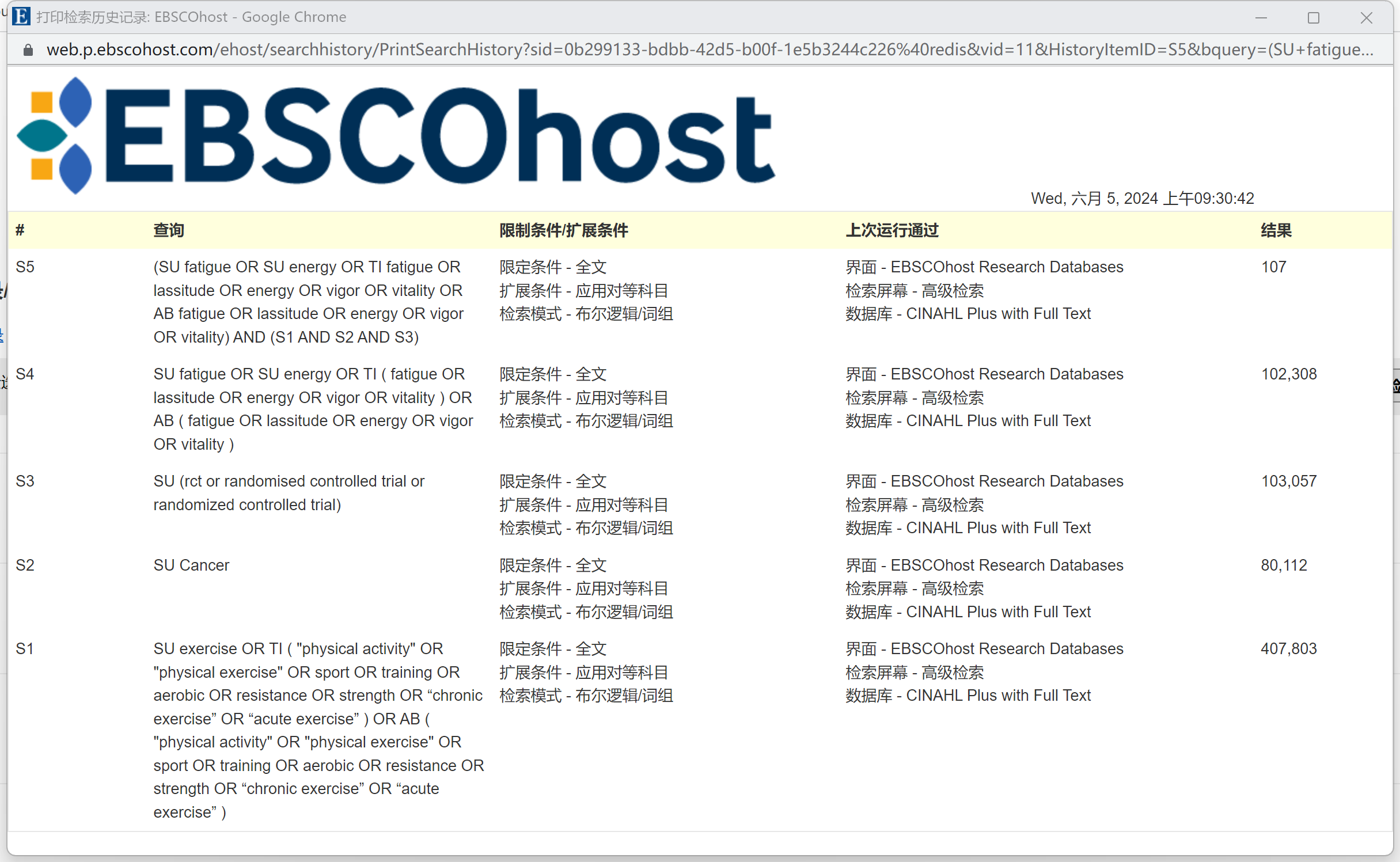

Supplement: S1 File — (ZIP) [file pone.0325100.s001.zip › Records retrieved for each database/ebsco.png]
